# Relationship between joint structure of the first tarsometatarsal joint and its degeneration

**DOI:** 10.1038/s41598-024-64064-x

**Published:** 2024-06-12

**Authors:** Kodai Sakamoto, Mutsuaki Edama, Haruki Osanami, Hirotake Yokota, Ryo Hirabayashi, Chie Sekine, Tomonobu Ishigaki, Hiroshi Akuzawa, Taku Toriumi, Ikuo Kageyama

**Affiliations:** 1 Institute for Human Movement and Medical Sciences, Niigata University of Health and Welfare, Shimami-cho 1398, Kita-Ku, Niigata, 950-3198 Japan; 2 Department of Anatomy, School of Life Dentistry at Niigata, Nippon Dental University, Niigata, Japan

**Keywords:** Anatomy, Musculoskeletal system, Bone, Musculoskeletal system, Bone

## Abstract

This study aimed to elucidate the relationship between joint structures of the first tarsometatarsal and articular facet degeneration. A total of 100 feet from 50 cadavers were examined. The articular facets of the first metatarsal and medial cuneiform were categorized into four types based on the superior and inferior facets' separation, and the formation of the inferior lateral facet on the lateral plantar prominence: Type I, a single facet with no separation or inferior lateral facet; Type II-a, two facets with separation but no inferior lateral facet; Type II-b, two facets, no separation, but with an inferior lateral facet; Type III, three facets with separation and an inferior lateral facet. When both bone types matched, they were defined as Type I, Type II-a, Type II-b, and Type III joints, respectively; unmatched types were classified as Unpair joints. The severity of articular cartilage degeneration on both bones was assessed using a 5-point scale. The degeneration grade was compared among joint types. Type III joints exhibited significantly milder articular cartilage degeneration in medial cuneiform compared to Type II-a, II-b, Unpair joints. The formation of inferior lateral facet and separation of the superior and inferior facets might be crucial for the joint's stability.

## Introduction

Hallux valgus is a common foot deformity that affects women more often than men^1,2^. The deformity in hallux valgus is characterized by valgus deviation of the proximal phalanx, pronation of the first metatarsal, and medial deviation of the first metatarsal head^2^. Hypermobility of the first tarsometatarsal joint (TMJ) is one factor associated with progression of hallux valgus^2^. Ellington et al. found TMJ hypermobility in 96% of patients with hallux valgus^3^. Moreover, a cadaver study showed that pronation of the first metatarsal increased the hallux valgus angle^4^. Control of TMJ hypermobility is thus crucial to prevent progression of hallux valgus.

The TMJ consists of the first metatarsal and the medial cuneiform. Singh et al.^5^ reported anatomical variations in the proximal articular facet of the first metatarsal, with some feet having a single facet and others having two distinct facets vertically, one superior and one inferior. Other studies compared the presence or absence of hallux valgus and TMJ mobility in the feet of single-facet and 2-facet types, finding no significant differences^6,7^. On the other hand, in research that examined 41 feet from formalin-fixed bodies, a 3-facet type, representing a foot with an inferior lateral articular surface on the lateral plantar prominence, was found^8^. According to that research, the single-facet type was only present in hallux valgus specimens and the 2-facet type was found in both normal and hallux valgus specimens, whereas the 3-facet type was exclusive to normal specimens^8^. In addition, a report using three-dimensional computed tomography suggested that the 3-facet type offers higher joint stability^9^. Consensus is thus lacking on the relationship between hallux valgus or joint stability and morphology of the articular surface at the base of the first metatarsal^6–9^. Furthermore, while the articular facet of the first metatarsal was extensively investigated, the distal articular facet of the medial cuneiform has not received the same level of scrutiny.

In addition, previous studies have reported that joint instability caused excessive incremental motion and uneven weight distribution, related to articular cartilage degeneration^10–12^. The structure of the TMJ may thus be associated with articular cartilage degeneration. However, the relationship between the joint structure of the TMJ and joint degeneration is unclear. In addition, although factors such as joint compatibility and sex also affect joint stability^13,14^, the joint compatibility and sex differences in joint surface morphology have yet to be clarified for the TMJ.

The purpose of this study was to clarify the relationship between the joint structure of the first metatarsal or medial cuneiform and articular cartilage degeneration of the TMJ. We hypothesized that the severity of articular cartilage degeneration in specimens with the 3-facet type of both first metatarsal and medial cuneiform would be milder than in that with other types.

## Results

### Reproducibility

The results of reproducibility for the Type classification of the first metatarsal and medial cuneiform were consistent between the Day 1 and Day 2. The grading of degeneration of the first metatarsal and medial cuneiform were also consistent between the Day 1 and Day 2. Cohen’s kappa coefficients for all measurements were 1.

### Type classification

Classifications of the first metatarsal and medial cuneiform are shown in Table 1. No significant differences were identified between males and females (first metatarsal: *P* = 0.60, medial cuneiform: *P* = 0.12) or between left and right sides (first metatarsal: *P* = 0.83, medial cuneiform: *P* = 0.27). Additionally, the joint type was described in Table 2. The combination of the first metatarsal and medial cuneiform types within Unpair joint was described in the Supplemental file (Table S1). No significant differences of age at death of specimens was found among joint type (*P* = 0.32).

**Table 1:** Comparison of classification of the first metatarsal bone and medial cuneiform by sex and laterality.

	First metatarsal bone				Medial cuneiform			
	Type I	Type II-a	Type II-b	Type III	Type I	Type II-a	Type II-b	Type III
Male (n = 56)	6 (11)	9 (16)	12 (21)	29 (52)	16 (29)	11 (20)	14 (25)	15 (27)
Female (n = 44)	3 (7)	12 (27)	9 (20)	20 (45)	9 (20)	7 (16)	6 (14)	22 (50)
Right (n = 50)	5 (10)	12 (24)	9 (18)	24 (48)	15 (30)	11 (22)	7 (14)	17 (34)
Left (n = 50)	4 (8)	9 (18)	12 (24)	25 (50)	10 (20)	7 (14)	13 (26)	20 (40)
Total (n = 100)	9 (9)	21 (21)	21 (21)	49 (49)	25 (25)	18 (18)	20 (20)	37 (37)

Values are reported as number of specimens (%).

**Table 2:** Prevalence of cartilage degeneration among the first tarsometatarsal joint type.

Joint type	N	Degeneration grade of first metatarsal bone					Degeneration grade of medial cuneiform bone				
		0	1	2	3	4	0	1	2	3	4
Type I	9	1	3	5	0	0	1	2	5	1	0
Type II-a	11	2	2	5	1	1	0	4	1	5	1
Type II-b	9	2	2	1	4	0	1	1	1	6	0
Type III	31	10	14	7	0	0	11	9	10	1	0
Unpair joint	40	11	12	10	6	1	7	8	13	9	3

Values are reported as number of specimens.

### Comparison of degeneration grade among joint types

There were no significant correlations between age at death of specimens and the degeneration grade of the first metatarsal (*r* = − 0.19*, P* = 0.95) and the medial cuneiform (*r* = − 0.2*2, P* = 0.79). The relationship between the degeneration grade in the first metatarsal and the medial cuneiform across different joint type is described in Tables 2 and 3. Regarding the degeneration grade in the first metatarsal, there was no main effect for joint type (*P* = 0.10). For the degeneration grade in the medial cuneiform, main effects were observed for joint type (*P* < 0.01). Post-hoc tests showed that Type III joints exhibited significantly lower articular cartilage degeneration in medial cuneiform compared to Type II-a (*P* = 0.03), II-b (*P* = 0.02), Unpair joints (*P* = 0.04).

**Table 3:** Comparison of cartilage degeneration of first metatarsal (MB1) or medial cuneiform (MCB) among joints types.

	Kruskal–Wallis test			Steel–Dwass		
	c2	df	*p*-value	Pair	W-statistics	*p*-value
First metatarsal degeneration grade	7.74	4	0.10	I versus II-a	0.81	0.98
				I verus II-b	1.04	0.94
				I verus III	− 2.64	0.33
				I verus Unpair	− 0.63	0.99
				II-a verus II-b	0.33	0.99
				II-a verus III	− 3.12	0.17
				II-a verus Unpair	− 1.36	0.87
				II-b verus III	− 2.7	0.31
				II-b verus Unpair	− .1.40	0.86
				III verus Unpair	2.25	0.5
Medial cuneiform degeneration grade	16.58	4	< 0.01	I verus II-a	1.74	0.74
				I verus II-b	2.43	0.42
				I verus III	− 2.53	0.38
				I verus Unpair	0.55	0.99
				II-a verus II-b	0.24	0.99
				II-a verus III	− 4.19	0.03
				II-a verus Unpair	− 1.55	0.81
				II-b verus III	− 4.25	0.02
				II-b verus Unpair	− 1.92	0.65
				III verus Unpair	3.99	0.04

### Comparison of degeneration grade with and without inferior lateral facet

The degeneration grade of the medial cuneiform was significantly higher in the joint without inferior lateral facet compared to the joint with inferior lateral facet (*Z* = − 1.71, *P* = 0.001). There was no significant difference in the degeneration grade of the first metatarsal with and without the inferior lateral facet (*Z* = − 3.21, *P* = 0.09).

## Discussion

The purpose of this study was to clarify the relationship between structure and degeneration of the TMJ. To the best of our knowledge, no anatomical studies have detailed the relationship between the structure of first metatarsal and medial cuneiform and articular cartilage degeneration of the TMJ.

For the first metatarsal, Type I was observed in 9%, Type II-a in 21%, Type II-b in 21%, and Type III in 49%. Wanivenhaus and Pretterklieber reported that the single-facet type was seen in 44% and the 2-facet type in 56%^15^. Doty et al. described that the single-facet and 2-facet types were seen in 59% and 41%, respectively^6^. However, these reports did not consider the inferior lateral facet, so Types II-b and III in our study were not included in their classification. On the other hand, Mason and Tanaka identified the single-facet type in 17%, the 2-facet type in 44%, and the 3-facet type in 39%^8^. Although classification methods differed, the 2-facet (Types II-a and II-b) and 3-facet types were more frequent than the single-facet type, suggesting that feet with 2 or 3 facets might be more common. In addition, racial differences have been identified in the angle of inclination of the first metatarsal proximal articular surface^16^. While race was not explicitly examined in previous studies^6,8,15^, the presence of racial differences in the number of articular surfaces might have contributed to the discrepancies observed between studies.

For the medial cuneiform, Type I was observed in 26%, Type II-a in 39%, Type II-b in 11%, and Type III in 24%. Ajmani et al. examined the articular facet of the medial cuneiform using the feet of 100 cadavers, with the single-facet type in 45% and the 2-facet type in 55%^17^. In addition, Dominick et al. also examined the articular facet of the medial cuneiform using the feet of 10 cadavers and reported single- and 2-facet types in 90% and 10%, respectively^18^. These reports classified the medial cuneiform based on the presence or absence of a constriction on the central articular surface. Hence, Types I and II-b in the present study are assumed to correspond to a single facet, and Types II-a and III to two facets. The present results therefore approximated the findings of Ajmani et al., that articular facets of the medial cuneiform with the constriction might be more frequent.

No sex differences were seen among types of either the first metatarsal or the medial cuneiform. A previous study reported that feet with one or three facets of the first metatarsal were more prevalent in males, while feet with two facets were more common in females^8^. One major reason for this inconsistency in findings is thought to be the small number of specimens, with 41 feet. The present study examined 100 feet, so the results were thought to offer a higher level of reliability than previous investigation.

The present study showed that the severity of articular cartilage degeneration in the medial cuneiform was lower for Type III joint than for Type II—a, Type III—b, Unpair joint. The morphological characteristics in Type III included the inferior lateral facet on the lateral plantar prominence and largest number of articular surfaces among joint types. In response to the former characteristics, Wanivenhaus et al. described that the lateral plantar prominence could play a role in preventing rotation of the first metatarsal^15^. Furthermore, reports using three-dimensional computed tomography have noted that joint stability in the sagittal plane is higher in feet with a high lateral plantar prominence^9^. In addition, prominences on the articular facet have been observed in other bones. For instance, the styloid process of the third metacarpal is thought to contribute to preventing hyperextension of the third metacarpal^19,20^. The bony stability provided by the lateral plantar prominence in the first metatarsal might therefore also play a crucial role in instability of the TMJ. However, the effect of the lateral plantar prominence on TMJ stability have yet to be empirically establish. Further research is required to elucidate the biomechanical significance of the lateral plantar prominence. Regarding the latter characteristics, it has been reported that the stability is higher for the type with a large number of joint surfaces that are separated in other joints. In the atlantoaxial joint, specimens with a single articular facet or with two articular facets have been found, and previous studies have suggested that the presence of two articular facets limited joint mobility^21,22^. Furthermore, in the subtalar joint, joints with a greater articular surface, specifically the 3-facet type, have been shown to exhibit greater joint stability^13,23^. The mechanism behind this was thought to be that separation of the articular surfaces causes the joint surface to have a more acute angle, thereby restricting movement^13,23^. Thus, individuals with separated articular facets of TMJ may thus also have a high degree of joint stability.

Several limitations need to be considered when interpreting the present findings. First, this study inferred joint instability from articular cartilage degeneration, so actual joint instability was not assessed. Further studies are required to ascertain the relationship between the joint structure, articular cartilage degeneration, and joint instability using biomechanical techniques. Second, the present study could not compare results between feet with hallux valgus and normal feet due to the difficulty of obtaining weight-bearing radiographs from formalin-fixed cadavers. Third, we were unable to examine the relationship between joint instability and ligaments or tendons surrounding the TMJ. Previous studies have indicated that the peroneus longus and plantar first tarsometatarsal ligament play important roles in joint stability^7,24^. Further studies are needed to clarify the relationship between the morphology of these musculotendinous unit or ligaments and joint stability. Fourth, this study did not evaluate the inter-rater reliability for evaluation of type classification and joint degeneration grade of medial cuneiform and first metatarsal.

## Methods

### Cadavers

This investigation examined 100 legs from 50 Japanese cadavers (mean age at death, 80 ± 11 years; range 64–105 years; 56 sides from men, 44 from women; 50 right sides, 50 left sides) that had been switched to alcohol after placement in 10% formalin. No feet showed any signs of previous major surgery around the foot. This study was conducted according to the guidelines of the Declaration of Helsinki. This study was approved by the ethics committee of the Niigata University of Health and Welfare (approval no. 19129–230,824). Informed consent for the storage and use of bodies for research purposes was given by donors before deaths or by their next of kin.

### Methods

The dissection procedure for the TMJ is described below. First, we sectioned the leg approximately 10 cm above the ankle joint, and isolated specimens of the leg were made. One examiner (KS) then removed the skin, subcutaneous tissue, and muscle by using forceps. Next, the same examiner dissected the surrounding soft tissues and joint capsule by using forceps, and exposed the articular surface of the TMJ by excising at the middle of the length of the plantar first tarsometatarsal ligament by using scissors. To avoid damage to the articular surfaces, care was taken during dissection to prevent the anatomical instruments from contacting the articular surfaces.

**Figure 1:**
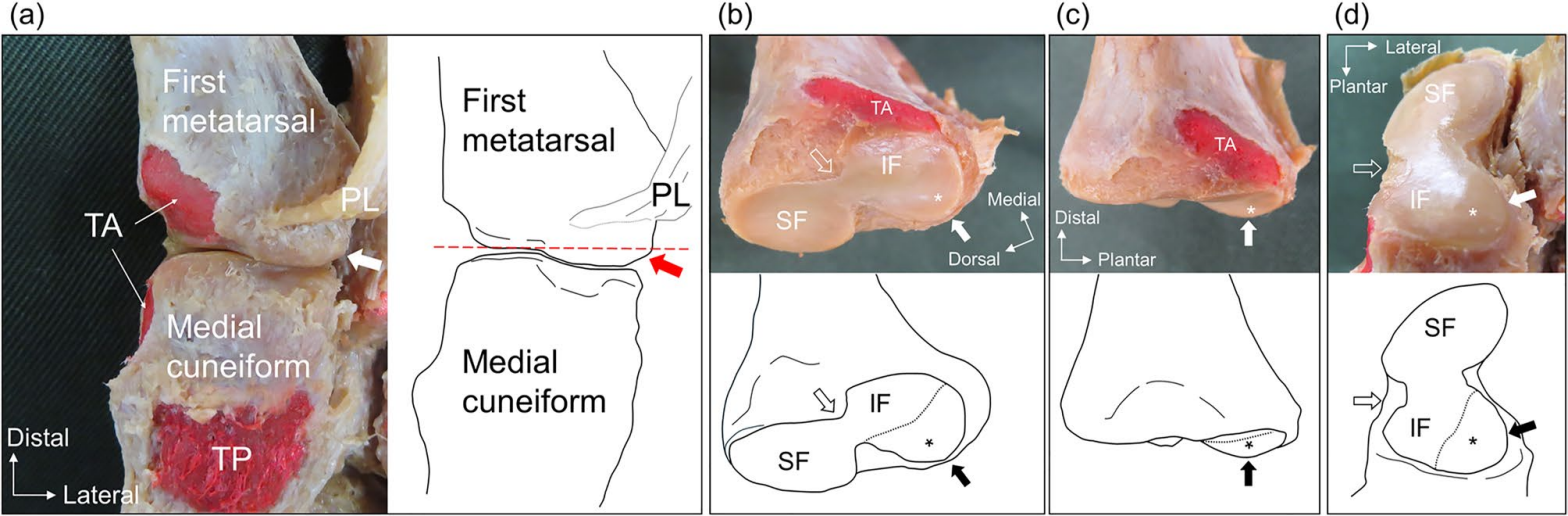
The structure of lateral plantar prominence in the first tarsometatarsal joint of left foot. (**a**) The plantar view of the first tarsometatarsal joint, and right image is the schematic illustration of this joint. The lateral plantar prominence (arrow) of first metatarsal is more protruded than medial articular surface. In (**b**, **c**, and **d**), the upper panel shows the anatomical photographs, and the lower panel shows the schema. (**b**) The medial plantar view of the proximal articular surface of the first metatarsal. The inferior lateral facet (asterisk) is formed on the lateral plantar prominence (arrow). (**c**) The medial view of the proximal articular surface of the first metatarsal. The inferior lateral facet (asterisk) is elevated from the inferior facet. The medial aspect of the articular surface has a constriction (blank arrow), which separates the superior and inferior facet. (**d**) The oblique plantar view of the medial cuneiform. The slope (arrow) is formed on the inferolateral surface of the medial cuneiform, which corresponds to the lateral plantar prominence of first metatarsal. The inferior lateral facet (asterisk) of medial cuneiform is formed on this slope. A constriction (blank arrow) on the medial aspect of the articular surface separates the superior and inferior facet. SF, superior facet; IF, inferior facet; PL, peroneus longus tendon; TA, attachment area of the tibialis anterior tendon; TP, attachment area of the tibialis posterior tendon.

**Figure 2:**
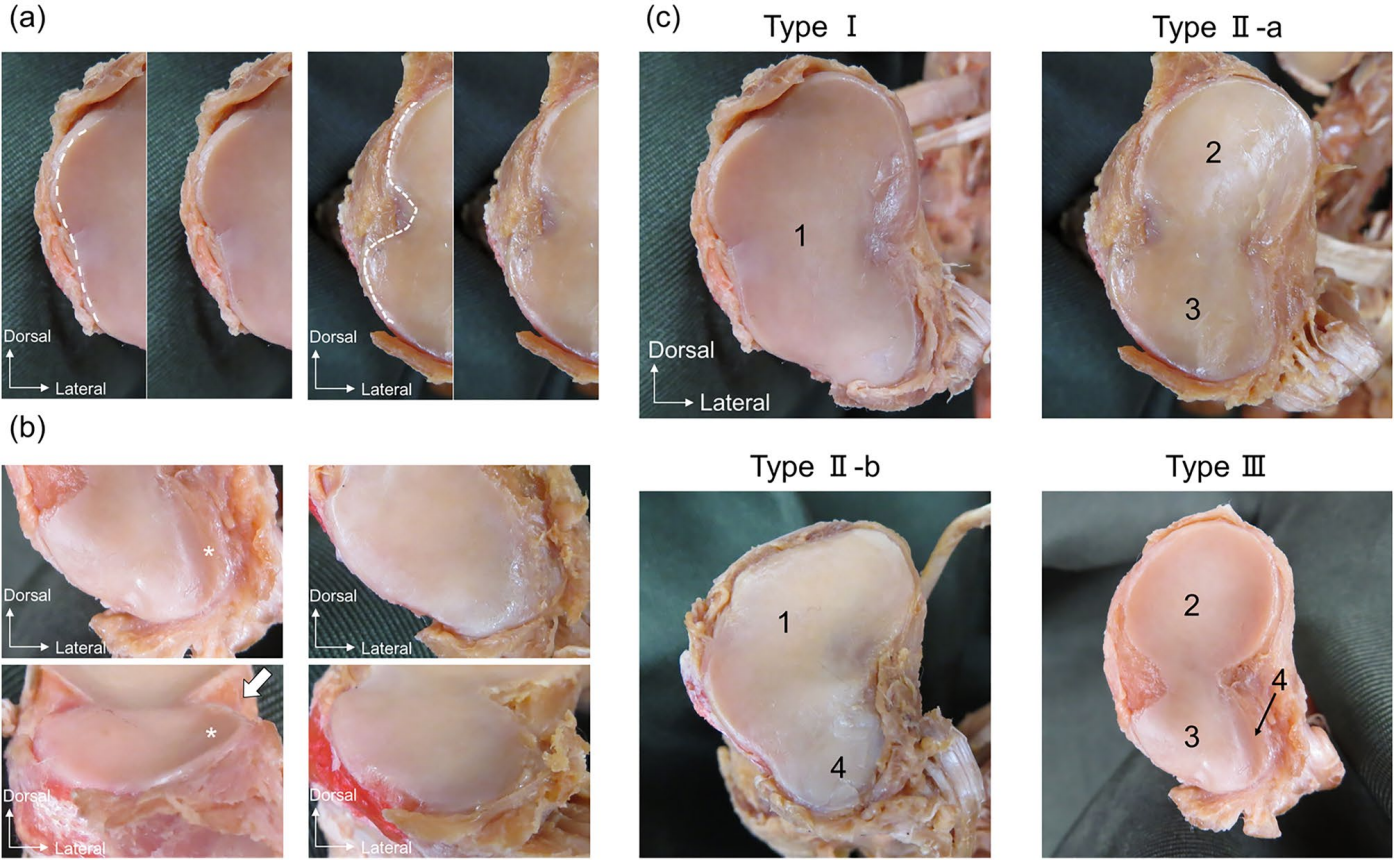
Procedure for classifying the articular surface of the first metatarsal bone; right foot. (**a**) Medial aspect of the articular surface of the first metatarsal. The left images show the articular surface without a separation, and the right images show the articular surface with a separation. (**b**) Plantar aspect of the articular surface of the first metatarsal. The left images show the articular surface with the inferior lateral facet (asterisk). Arrow point to lateral plantar prominence. The right images show the articular surface without an inferior lateral facet. (**c**) Type classification of the first metatarsal. 1, a facet not divided into superior and inferior; 2, superior facet; 3, inferior facet; 4, inferior lateral facet.

**Figure 3:**
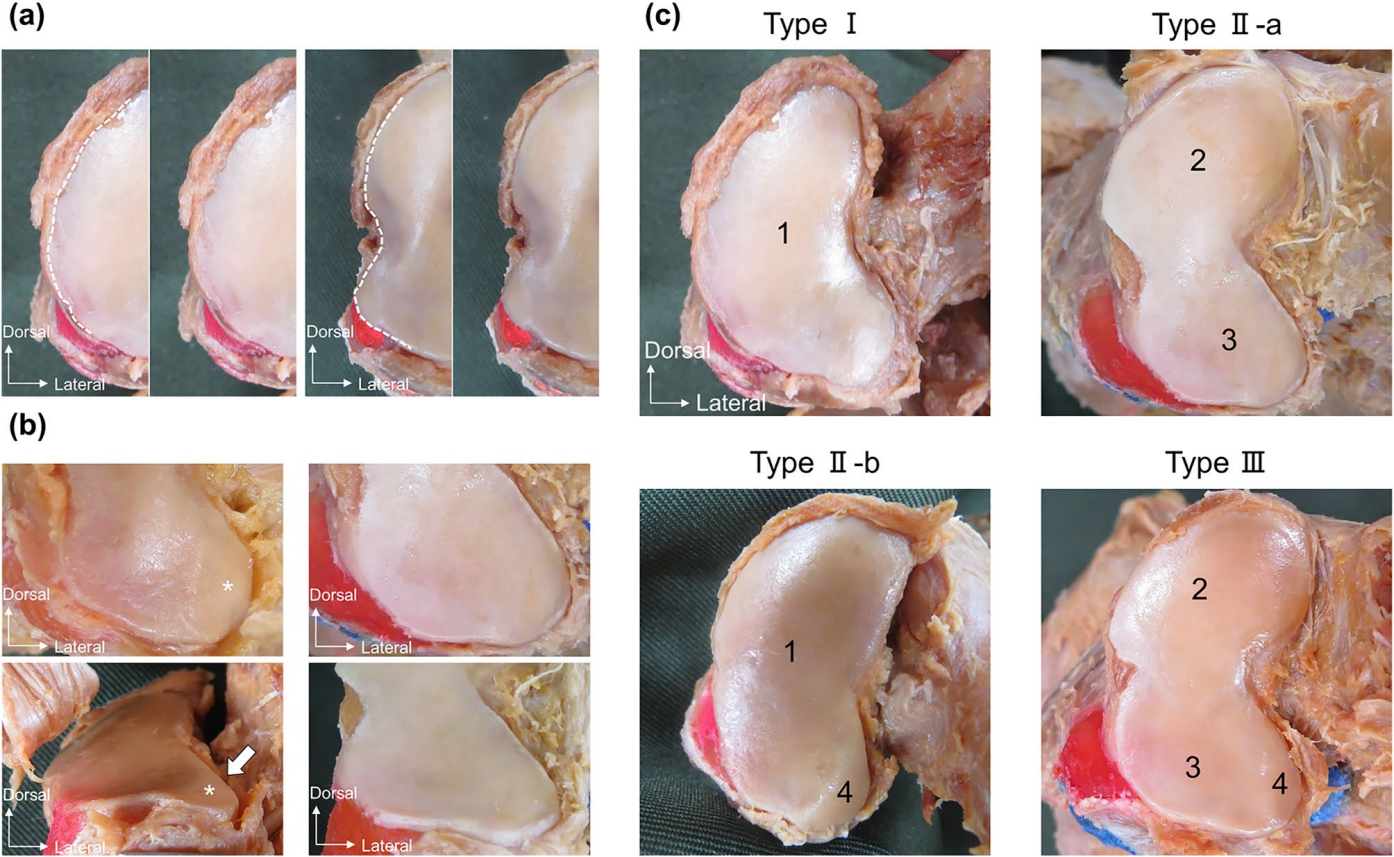
Procedure for classifying the articular surface of the medial cuneiform bone; left foot. (**a**) Medial aspect of the articular surface of the medial cuneiform. The left images show the articular surface without separation, and the right images show the articular surface with separation. (**b**) Plantar aspect of the articular surface of the medial cuneiform. The left images show the articular surface with an inferior lateral facet (asterisk). Arrow point to the slope at the inferolateral aspect of the articular surface. The right image shows the articular surface without an inferior lateral facet. (**c**) Type classification of the medial cuneiform. 1, a facet not divided into superior and inferior; 2, superior facet; 3, inferior facet; 4, inferior lateral facet.

The bony morphology of the first tarsometatarsal joint was described in Fig. 1. In both first metatarsal and medial cuneiform, separation of the superior and inferior facets was identified based on the presence or absence of a constriction on the medial aspect of the articular surface (Fig. 1c,d). A bony prominence can be observed in the inferolateral aspect of the first metatarsal base, which is more prominence towards the articular surface than the inferomedial aspect of the first metatarsal (Fig. 1a–c). In reference to previous studies^8,9,15^, the bony structure found on the inferolateral side of the first metatarsal base, protruding more than the medial articular surface, was identified as the lateral plantar prominence in this study. The lateral plantar prominence creates an angled slope on the lateral part of the inferior articular surface of the first metatarsal, separating part of the inferior articular surface. This separated articular surface is referred to as the inferior lateral facet (Fig. 1a–c). Conversely, the lateral plantar prominence is absent or is considered negligibly low and not viewed as a protrusion, the inferior lateral facet did not form. Similar to the first metatarsal, the articular facet located on the slope at the inferolateral aspect of the articular surface of the medial cuneiform, which corresponds to the lateral plantar prominence of the first metatarsal, was defined as the inferior lateral facet (Fig. 1d). Based on these standards, the articular facets of the first metatarsal and the medial cuneiform were classified into 4 types, respectively (Figs. 2 and 3). Type I was a single facet without separation of the superior and inferior facets and with no inferior lateral facet. Type II-a was two facets with separation of the superior and inferior facets and with no inferior lateral facet. Type II-b was two facets without separation of the superior and inferior facets and with an inferior lateral facet. Type III was three facets with separation of the superior and inferior facets and with an inferior lateral facet. Next, the joint type was classified based on the types of both first metatarsal and medial cuneiform for evaluation of joint compatibility. Specifically, those with congruent articular surface types of the first metatarsal and medial cuneiform were classified as the corresponding joint type (e.g., if both the first metatarsal and medial cuneiform were Type I, they were classified as Type I joints). Contrary, those without congruency were classified as the “Unpair joint”.

**Figure 4:**
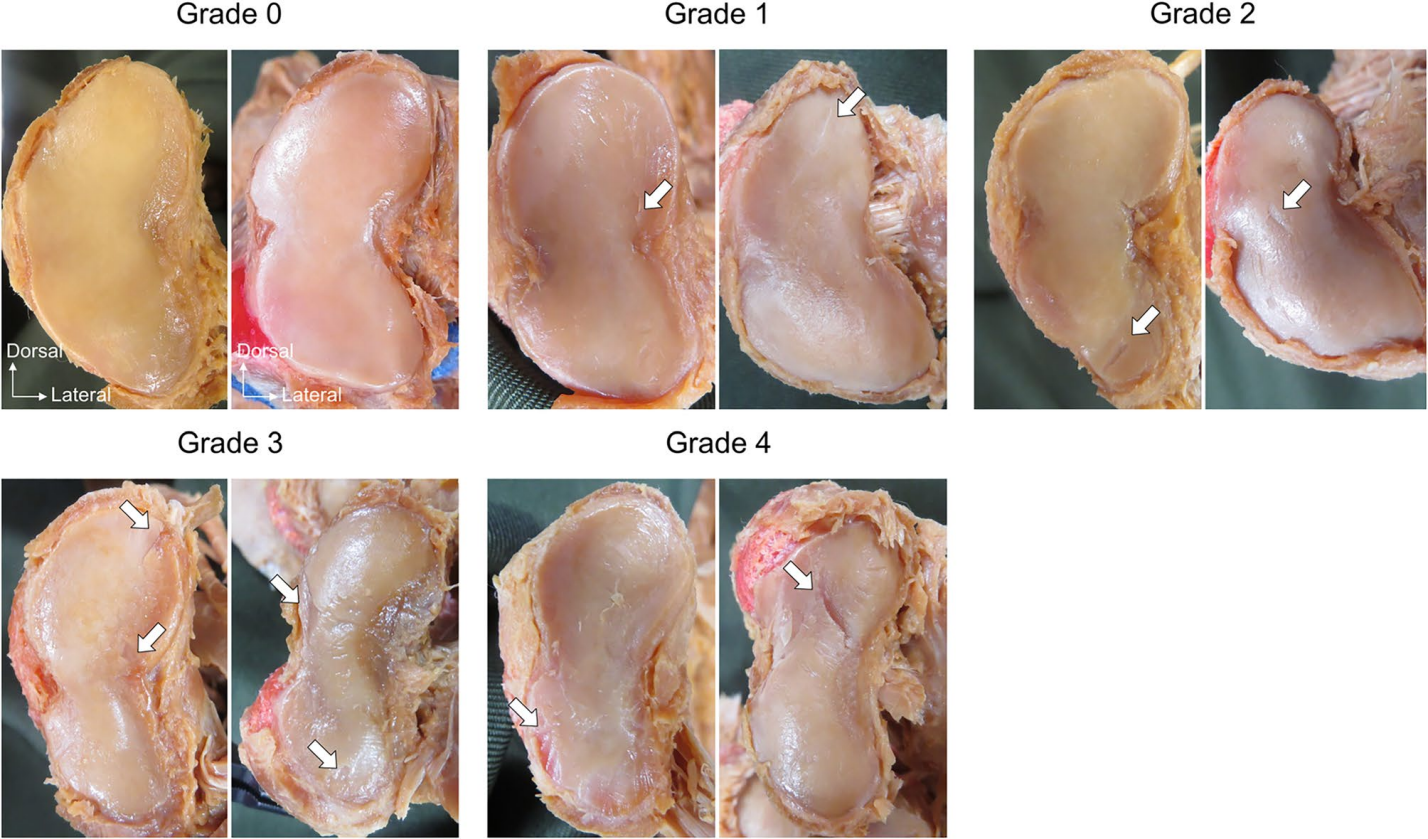
Procedure for classifying grade of degeneration of first metatarsal and medial cuneiform articular facets. The left side shows the articular surface of the first metatarsal, and the right side shows the articular surface of the medial cuneiform. Arrow, areas showing degeneration.

The severity of articular cartilage degeneration of the first metatarsal and medial cuneiform was graded using the International Cartilage Repair Society scale as reference^25^ and the following independent criteria: Grade 0, absence of cartilage damage; Grade 1, presence of cracks in the cartilage surface; Grade 2, degeneration observed in the superficial layer of cartilage; Grade 3, degeneration reaching the deep cartilage layer where the subchondral bone was visible through the articular cartilage; Grade 4, degeneration reaching the subchondral bone or deeper (Fig. 4). The type classification of the first metatarsal and medial cuneiform and the grading of articular cartilage degeneration were determined again using the same method within 1 week but at least two days after the initial determination, to evaluate reproducibility.

### Statistical analysis

Statistical analyses were performed using R statistical software (R Foundation for Statistical Computing, Vienna, Austria). To assess the reproducibility of articular surface type classification and grading of articular cartilage degeneration, we calculated Cohen’s kappa coefficient for both the joint surface type classification and the grading of cartilage degeneration based on evaluations from Day 1 and Day 2. Fisher’s exact test was used for comparisons between male and female specimens and between left and right feet for the first metatarsal and medial cuneiform types. The Kruskal–Wallis test was used for comparison of age at death of specimens among joint type. The relationship between age at death of specimens and the degeneration grade of the first metatarsal and medial cuneiform were assessed by Spearman’s rank correlation coefficient. The degeneration grade of the first metatarsal and medial cuneiform were compared among joint type using the Kruskal–Wallis test. Additionally, Mann–Whitney U test was used to compare the degeneration grade of the first metatarsal and medial cuneiform with and without the inferior lateral facet. Differences were considered significant at the level of *p* < 0.05.

### Ethics approval and consent to participate

This study was approved by the ethics committee of the Niigata University of Health and Welfare (approval no. 19129–230,824). Informed consent for the storage and use of the bodies for research purposes was given by the donors prior to their deaths or by their next of kin.

## Conclusion

In the present study, the first metatarsal and medial cuneiform were each classified and related to articular cartilage degeneration. The formation of the inferior lateral facet and separation of the superior and inferior facets may thus represent important factors in stability of the TMJ. Future studies should investigate in detail relationships between morphologies of the ligaments and musculotendinous units surrounding the TMJ.

### Supplementary Information

Supplementary Information.

## Data Availability

The data and the corresponding statistical analyses of this study are available upon request from the corresponding author.
