# Supplementary material for: Relationship between joint structure of the first tarsometatarsal joint and its degeneration
Source: Sci Rep. 2024 Jun 12;14:13547. doi: 10.1038/s41598-024-64064-x (PMC11169535; doi:10.1038/s41598-024-64064-x)
Supplement: Supplementary file 1 — Supplementary Information. [file 41598_2024_64064_MOESM1_ESM.pdf]

Title:

Relationship between joint structure of the first tarsometatarsal joint and its degeneration.

Authors:

Kodai Sakamoto[1*], Mutsuaki Edama[1,2], Haruki Osanami[1], Hirotake Yokota[1], Ryo Hirabayashi[1], Chie Sekine[1], Tomonobu Ishigaki[1], Hiroshi Akuzawa[1], Taku Toriumi[2], Ikuo Kageyama[2]

Affiliations:

[1] Institute for Human Movement and Medical Sciences, Niigata University of Health and Welfare, Niigata, Japan

[2] Department of Anatomy, School of Life Dentistry at Niigata, Nippon Dental University, Niigata, Japan

Table S1 The combination of the first metatarsal and medial cuneiform types within Unpair joint

	First metatarsal type	Medial cuneiform type	Number	%
Pair 1	Ⅱ-a	Ⅰ	8	20.0%
Pair 2	Ⅱ-a	Ⅲ	2	5.0%
Pair 3	Ⅱ-b	Ⅰ	4	10.0%
Pair 4	Ⅱ-b	Ⅱ-a	4	10.0%
Pair 5	Ⅱ-b	Ⅲ	4	10.0%
Pair 6	Ⅲ	Ⅰ	4	10.0%
Pair 7	Ⅲ	Ⅱ-a	3	7.5%
Pair 8	Ⅲ	Ⅱ-b	11	27.5%
Total	-	-	40	-
